# Using Portable Force Plates to Assess Vertical Jump Performance: A Metrological Appraisal

**DOI:** 10.3390/sports6040149

**Published:** 2018-11-19

**Authors:** François Raymond, Benoit Lussier, François Dugas, Mathieu Charbonneau, Félix Croteau, Cory Kennedy, Nicolas Berryman

**Affiliations:** 1 Institut National du Sport du Québec, 4141 Pierre de Coubertin, Montreal, QC H1V 3N7, Canada; fraymond@insquebec.org (F.R.); blussier@insquebec.org (B.L.); francois.dugas@outlook.fr (F.D.); mcharbonneau@insquebec.org (M.C.); fcroteau@insquebec.org (F.C.); ckennedy@insquebec.org (C.K.); 2 School of Physical and Occupational Therapy, McGill University, 3654 Sir William Osler, Montreal, QC H3G 1Y5, Canada; 3 Department of Sports Studies, Bishop’s University, 2600 College, Sherbrooke, QC J1M 1Z7, Canada

**Keywords:** neuromuscular monitoring, validity, reliability

## Abstract

The purpose of this study was to verify the metrological properties of portable force plates that are used to assess countermovement jump performance. While 88 participants (38 males, 50 females) were included in the agreement analyses, 84 participants (37 males and 47 females) completed the reliability part of the study. This randomized crossover design suggests that portable force plates could be used interchangeably with a reference system. Indeed, the differences between both devices were all considered trivial (effect size (ES) < 0.20), and the mean bias was never greater than 3.41% in comparison to the reference system. In addition, the absolute and relative reliability parameters were found to be acceptable for clinical use, even when used on different floor surfaces. However, it was found that the ratio between flight time and contraction time (FTCT) showed questionable reliability when tests were conducted on different surfaces (intraclass correlation coefficient = 0.49; coefficient of variation = 26.72%). Therefore, practitioners should be careful when installing the portable device on different floor surfaces in order to optimize the reliability and the ability to detect real change in the context of a countermovement jump monitoring process.

## 1. Introduction

The Goldilocks’ principle appropriately describes the relationship between training load and performance, which suggest that the “right amount” of training must be established [1]. Moreover, since the response to training is highly individual [2], a single training recipe can’t be applied to every athlete. Therefore, a sound monitoring strategy should be implemented in order to optimize sports performance on an individual basis. Such a monitoring program will allow the sport coaches and the integrated support team to find the appropriate balance between training and recovery, which should prevent negative adaptations such as overreaching and injuries [3]. 

In order to monitor athletes, many data coming from a wide variety of sources are available for the practitioner (heart rate, rate of perceived exertion, psychometric questionnaires, performance variables, etc.) [4]. In the last decade, the assessment of neuromuscular function via countermovement jump testing received a lot of attention [5,6]. While this approach can be used for the formal assessment of jumping performance [7] or with an injury prevention motive [8], practitioners have also tried to implement a recurring assessment of jumping performance to gain insight into the interplay between training and recovery [9,10]. Such an approach has been shown to provide valid information on neuromuscular function and training readiness [11], while also being sensitive enough to respond to increased training loads [5]. Consequently, it has empowered strength and conditioning practitioners, scientists, and sport coaches by providing valuable information on fitness–fatigue paradigms and by helping to further individualize the training stimulus [12]. 

Multiple devices are available to assess jump performance, but force platforms definitely provide advantages. Indeed, knowing jump height is not always a reliable metric of an athlete’s recovery and current readiness. Force platforms provide the ability to understand the mechanisms underpinning jump height, not just the final outcome of the movement itself. This feature seems to be crucial to practitioners interested in athletes’ monitoring [6]. Given their size, weight, and the specialized equipment necessary for their proper use, traditional force platforms have generally been constrained to the daily training environment (DTE) in high-performance facilities. While representing a key piece of the monitoring process when the athletes are in the DTE, their expensive costs and limited portability threaten the monitoring process. Indeed, as soon as the athletes are away for camps or competitions, members of the support staff lose this precious stream of information. Whether it is for training camps, staging camps, or competitions, moments away from the DTE represent crucial moments in terms of decision-making about the appropriate training and recovery balance that is associated with optimal adaptations and performance. Since the need for high-quality monitoring information is elevated in such a context, being able to maintain all the data streams as in the DTE would represent a clear competitive advantage. Consequently, there is a strong demand for a valid and reliable monitoring tool that would mitigate the absence of traditional force platforms outside of the DTE. 

Recent technological developments have given sport scientists a chance to circumvent the aforementioned predicament. Namely, light and portable force plates are now available, and could provide an interesting alternative to coaches looking to export their monitoring strategy out of the DTE. However, to our knowledge, the metrological parameters of these devices still need to be established. While the validity was recently verified in a sample of 28 men [13], reliability when different floor surfaces are used has never been studied. Considering its potential usage while traveling for training camps and in the last stages before competition, it appears that quantifying this metrological property is important. Therefore, the first objective of this study was to confirm the portable force plates’ validity by investigating the level of agreement between portable and fixed force plates, which will determine whether both systems could be used interchangeably in the DTE [14]. In addition, as practitioners are likely to travel in a variety of environments with this portable equipment, the second objective of this study was to determine the reliability parameters when floor surfaces are modified. Our hypotheses were that portable force plates would display high validity when compared to a reference system. However, we expected that changes in the floor surface would alter the portable force plates’ reliability. 

## 2. Materials and Methods

### 2.1. Participants

Participants, being regular visitors of the strength and conditioning room, were recruited through posters installed in the training facility. National and provincial team athletes along with trained members of the staff above the age of 18 were recruited. All of the work was conducted with the formal approval of the Research Ethics Board from the corresponding author’s university affiliation, and participants were informed of the benefits and risks of the investigation prior to signing a consent form. Eighty-eight participants (38 males and 50 females) were included in the interchangeability subsection (mean ± SD; age: 24.72 ± 6.52 years; body mass: 72.08 ± 12.47 kg). Secondly, the reliability part included 84 participants (37 males and 47 females). Mean age and body mass were (mean ± SD): 24.50 ± 6.34 years and 72.67 ± 12.46 kg. Participants’ descriptive characteristics are presented in Table 1. 

### 2.2. Experimental Approach to the Problem

This study was a randomized crossover design with three sessions to complete the protocol. After a first session during which participants were familiarized with the testing conditions (jumps made on each device), a second session was dedicated to the interchangeability question, while reliability was addressed during a third session. A delay of at least 24 h separated each of the three sessions. 

To evaluate the interchangeability of the equipment, participants were asked to perform three maximal countermovement jumps (CMJ) on a portable (PORT) device (PASPORT PS 2141—35 cm × 35 cm, PASCO, Roseville, CA, USA) and a reference (REF) system (BP600600-1000—60 cm × 60 cm, Advanced Medical Technologies Inc., Watertown, MA, USA). The REF system was resting directly on rubber flooring, while each of its four anchors were going through the superficial layer to sit on a concrete surface. Participants followed randomly one of the two possible sequences (REF–PORT or the opposite). A standardized warm-up consisting of 10 body weight squats, 10 body weight walking lunges, five progressive body weight squat jumps, and three maximal body weight CMJs were performed before the data collection. There was a two-minute rest period after the warm-up, 15 s of rest between each of the three jumps, and a five-minute break between the two conditions (PORT or REF). Participants were asked to jump as high as possible on each attempt. The CMJ posture was standardized with the arms akimbo, and countermovement depth was not controlled.

During the third testing session, portable device’s reliability was assessed when modifications were made to the underneath floor surfaces. Participants randomly followed one of the two possible sequences (strength and conditioning room [S&C]–laboratory [LAB] or the opposite), and three maximal jumps were made for each condition. These rooms were chosen because of the key differences in their respective floor properties. While the floor of the S&C room was made of rubber mats, the force plates were positioned directly on the concrete surface of our training facility’s LAB. Warm-up and jump instructions for this session were identical to the second session. A five-minute break was necessary between conditions to allow moving and reinstalling the equipment in the second room of the sequence. 

### 2.3. Data Analyses

The PORT platforms were interfaced, by default, to a Capstone software. Raw data were then processed with another custom-made MatLab script (MathWorks, Natick, MA, USA). The same custom-made MatLab script was also used for the REF system. All of the trials were collected at a sampling frequency of 1000 Hz. Furthermore, an oversampling procedure was conducted to reach a sampling of 10,000 Hz to allow the precise detection of jumping events during signal processing procedures. For each of the conditions (REF versus PORT and LAB versus S&C), the average of the three jumps was used for analysis. Based on their relevance in jump monitoring and neuromuscular readiness assessment, five variables were selected and included in our analyses.

*Maximal Force* (*FMax*)*:* This variable represents the maximal vertical force produced by the participant during the jump between the onset of movement and take-off. The onset of the countermovement was defined as the moment when the force–time curve moved three standard deviations away from the body mass value that was measured while the athlete was quietly standing on the force plates. As for the take-off threshold, it was set as the first zero or the first negative value on the force–time curve, whichever came first. 

*Time to reach maximal force* (*TFMax*)*:* This marker combines temporal and vertical force data to describe the amount of time, from the onset of the jump, to reach the maximal amount of vertical force. While it usually coincides with the transition from the braking to the concentric portion of the jump, it can also match the maximal concentric force for certain types of jumpers. 

*Rate of Force Development* (*RFD*)*:* RFD represents, on the force–time curve, the slope between the lowest force produced (immediately before the onset of the braking phase, during the unweighting phase) and the peak force measured before the take off. 

*Impulse* (*IMP*): This variable is based on the impulse–momentum relationship. We used the net vertical impulse in our calculations by taking into account the impulse–moment theorem, the center of mass’ velocity and the athletes’ body mass [15]. Both the braking and propulsive phases were integrated to determine the net impulse. 

*Flight Time/Contraction Time* (*FTCT*)*:* This ratio between flight time and contraction time provides insight into the effectiveness of a jump. Contraction time starts at the onset of the jump and finishes at take-off, while flight time is self-explanatory. 

### 2.4. Statistical Analyses

For the second and third session, the average of all three maximal jumps executed was used for further analyses. Standard procedures were used to calculate descriptive statistics (means, standard deviations, and frequencies). Data were considered as outliers if they were more than 2.5 standard deviations (SD) away from the mean. In such a case, outliers were replaced by a score corresponding to the mean + 2.5 SD. This procedure has the advantage of helping normalize the data distribution while maintaining the relative position of a score in the sample [16,17]. A Shapiro–Wilk test was conducted to assess that all of the variables were normally distributed, and a Levene’s test was used to verify the homogeneity of the variance. For both interchangeability and reliability analyses, paired *t*-tests were carried out when data met this normality assumption, whereas a Wilcoxon test was used for non-normally distributed variables. Effect sizes (ES) were then calculated using procedures described elsewhere [18], and Cohen’s scale was used to interpret each ES (ES < 0.2 = trivial, 0.2 ≤ ES < 0.5 = small, 0.5 ≤ ES < 0.8 = moderate, ES > 0.8 = large) [19]. The level of agreement between REF and PORT was assessed with Bland Altman plots, which helped determine if two systems could be used interchangeably [14,20,21]. Intraclass correlation coefficients (ICC), standard error of measurements (SEM), coefficients of variation (CV, standard deviation of the differences between conditions divided by the mean of all observations × 100) and minimum differences to be considered real (MD) were calculated to verify reliability when different floor surfaces are used underneath the PORT device [22,23]. Statistical analyses were completed with SPSS (IBM SPSS Statistics, Version 24), and the level of significance was established at *p* < 0.05. Bland Altman graphs were exported from MedCalc (MedCalc Statistical Software version 18.6).

## 3. Results

After exploring for potential outliers, it was found that approximately 2% of the values of the grand total of jumps performed were more than 2.5 SD away from the mean, which resulted in a modification as described in the methods section. 

### 3.1. Interchangeability

**Figure 1:**
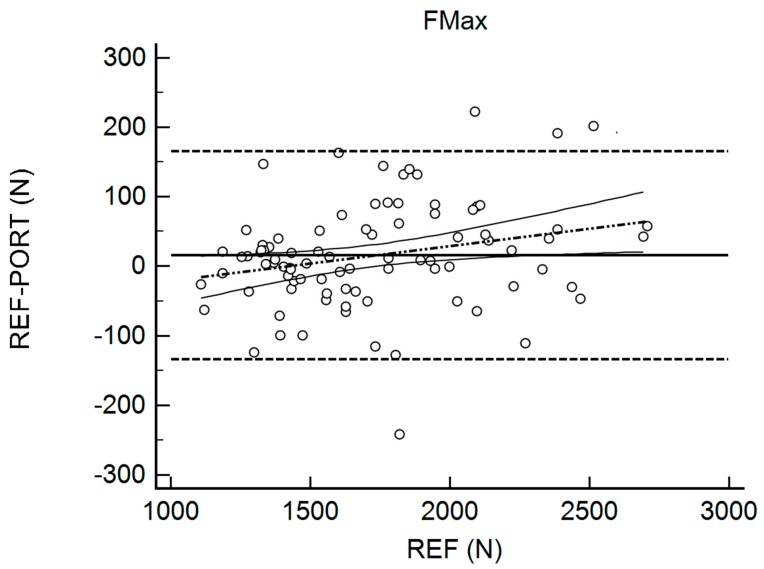
Bland Altman Plot for Maximal Force (FMax). The horizontal continuous line represents the mean bias, whereas the horizontal dashed lines represent the 95% limits of agreement. The discontinuous dashed line and the associated curvilinear continuous lines represent the regression and its 95% confidence intervals.

**Figure 2:**
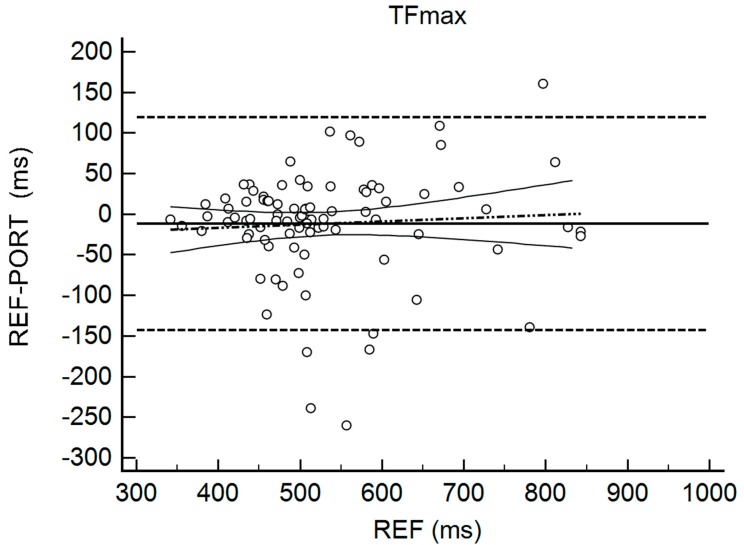
Bland Altman Plot for Time to Reach Maximal Force (TFMax). The horizontal continuous line represents the mean bias, whereas the horizontal dashed lines represent the 95% limits of agreement. The discontinuous dashed line and the associated curvilinear continuous lines represent the regression and its 95% confidence intervals.

**Figure 3:**
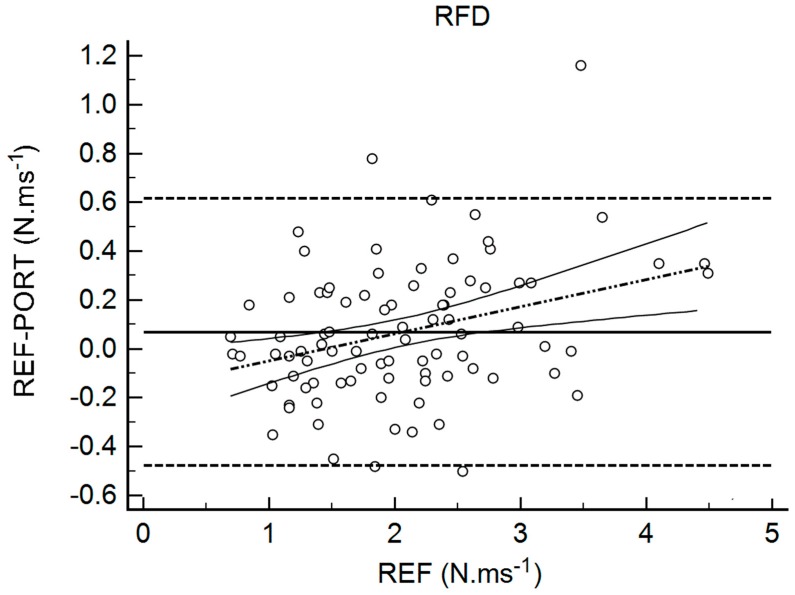
Bland Altman Plot for Rate of Force Development (RFD). The horizontal continuous line represents the mean bias, whereas the horizontal dashed lines represent the 95% limits of agreement. The discontinuous dashed line and the associated curvilinear continuous lines represent the regression and its 95% confidence intervals.

**Figure 4:**
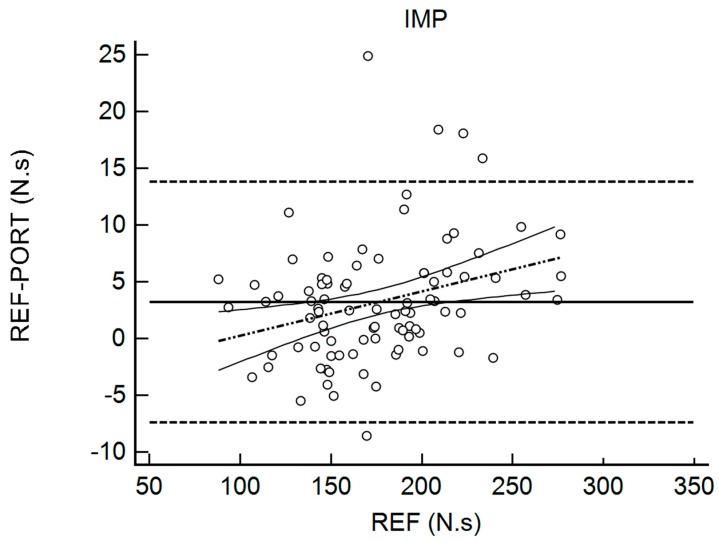
Bland Altman Plot for Impulse (IMP). The horizontal continuous line represents the mean bias, whereas the horizontal dashed lines represent the 95% limits of agreement. The discontinuous dashed line and the associated curvilinear continuous lines represent the regression and its 95% confidence intervals.

**Figure 5:**
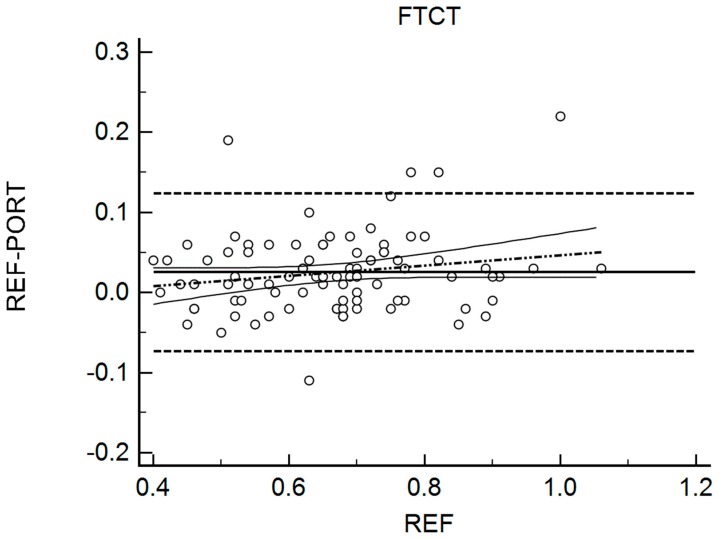
Bland Altman Plot for Flight Time/Contraction Time (FTCT). The horizontal continuous line represents the mean bias, whereas the horizontal dashed lines represent the 95% limits of agreement. The discontinuous dashed line and the associated curvilinear continuous lines represent the regression and its 95% confidence intervals.

Table 2 shows the results for the interchangeability analysis. Significant differences (*p* < 0.05) were found between testing devices for IMP and FTCT. When looking at the ES values, all of the differences were considered trivial (−0.10 < g < 0.17). The bias (±95% limits of agreement, LOA) was 15.45 N (150.59) for FMax, −11.69 ms (131.90) for TFMax, 0.07 N·ms^−1^ (0.55) for RFD, 3.19 N·s (10.66) for IMP, and 0.02 (0.10) for FTCT. Figure 1, Figure 2, Figure 3, Figure 4 and Figure 5 are showing the LOA between the testing devices for each variable.

### 3.2. Reliability

Table 3 shows the results for the reliability analysis. No significant differences were found between testing environments (S&C—rubber flooring versus LAB—concrete flooring). When looking at the ES values, all of the differences were considered trivial (−0.01 < g < 0.11). ICC values were within a range from 0.48 to 1.00, whereas SEM represented between 1.73% to 19.08% of the mean values. The CVs ranged from 2.45% to 26.72% and the MD, which was expressed as a percentage of the highest mean, and varied between 4.80–53.23%.

## 4. Discussion

The objective of this study was to assess the metrological properties of portable force platforms during countermovement jumps. Our hypotheses were that portable force plates would display high similarities when compared to a reference system, which would allow using both systems interchangeably. However, we expected that the reliability parameters would not be acceptable for monitoring purposes when the tests were conducted on two different floor surfaces. 

Regarding the interchangeability with a reference system, the results revealed that significant statistical differences could be observed for two of the five variables included in our analyses (IMP and FTCT), whereas two more were really close to reach the significance threshold (FMax, TFmax). Interestingly, a tendency toward better performance measured by the REF system was observed for all of the variables. While it is difficult to identify a definitive cause for this phenomenon with the actual data set, differences between PORT and REF could be partly explained by a variability in jump performances, since different jumps were made on both devices. Nonetheless, when analyzing the mean bias observed between the two methods, it was found that it never represented more than 3.41% of the mean values reported by the REF system. In addition, an analysis of the effects sizes showed that these differences were considered trivial. Notably, these results are in line with a previous study despite methodological differences. Indeed, in the study by Lake et al., participants jumped on portable devices installed on top of the reference system, allowing for simultaneous data collection [13]. Taken together, these results obtained here with a larger sample constituted of both men and women tend to confirm that both devices could be used interchangeably. 

Considering that one key advantage of the PORT system is related to the possibility to travel with the device in different training and competition environments, it appeared quite important to assess its reliability when different floor surfaces are used. In our study, the PORT system was positioned directly on a concrete surface and on a floor made of rubber mats, which could represent typical differences when traveling with athletes. When comparisons were made between the two environments, no statistical differences were found, and the effect sizes were all trivial. In addition, we assessed both the relative and absolute reliability. While the ICC indicates if an individual maintains his position in comparison to the group during repeated measurements, the SEM and the CV could be used to assess the variability in measures repeated over time [22,23]. In this study, the ICC of four variables was above 0.8, with values below this threshold being considered questionable for clinical use [24]. Notably, three variables (FMax, RFD, IMP) reached ICC values greater than 0.9, which is considered a high relative reliability score [24]. However, it seems that the FTCT ratio is more variable with an ICC of only 0.48. In line with this observation, the CV of this ratio is by far the largest of the variables included in our analyses, with a value (26.72%) representing more than double that of the second-largest CV (13.07; IMP). Taken together, these results suggest that the FTCT ratio is showing a questionable reliability, which could have important consequences considering that this variable seems pertinent in a monitoring strategy [5]. Whether the floor surface is playing a role or not in the observed differences still needs to be confirmed, but these results suggest that practitioners should, at least, be really careful about the installation of the PORT device when traveling with the equipment. Importantly, when looking at the minimum difference to be considered real (MD), which could be considered as the upper limit of the noise in the measurement, the value computed for the FTCT ratio represented 53.23% of the mean score when compared with the highest mean. These values were under 10% for FMax and IMP, and this analysis reinforces that the practitioner should strictly control testing conditions in order to optimize reliability and, therefore, the ability to detect real change.

## 5. Practical Applications

The objective of this study was to assess the metrological properties of portable force platforms. Our analyses revealed a good level of agreement between the portable and the reference devices included in this study, which suggests that these systems could be used interchangeably. In addition, it was found that, for most of the variables, the reliability parameters were acceptable for clinical use when tests were conducted on different mounting surfaces. Nevertheless, practitioners should be careful when installing the portable device on different floor surfaces in order to optimize reliability and the ability to detect real change. 

## Figures and Tables

**Table 1:** Participants’ descriptive characteristics.

Variables	Interchangeability Analysis	Reliability Analysis
Cohort size	88	84
Sex	38 M, 50 W	37 M, 47 W
Testing sequence	43 PORT-REF, 45 REF-PORT	40 GL, 44 LG
Age ^a^	24.72 (6.52)	24.50 (6.34)
Body mass ^b^	72.08 (12.47)	72.67 (12.46)

M = Men, W = Women, PORT = Portable, REF = Reference, G = Gym, L = Lab. ^a^ in years old (standard deviation); ^b^ in kg (standard deviation).

**Table 2:** Interchangeability parameters.

Variables	Portable	Reference	*p* Value	ES	Bias (±95% LOA)	Bias (%REF)
FMax (N)	1710.24 (370.31)	1725.69 (381.93)	0.060	0.04	15.46 (149.74)	0.90
TFMax (ms)	541.87 (125.76)	530.17 (110.85)	0.327	−0.10	−11.69 (131.16)	−2.20
RFD (N·ms^−1^)	1.98 (0.77)	2.05 (0.82)	0.060	0.08	0.07 (0.55)	3.41
IMP * (N·s)	172.11 (40.09)	175.30 (41.37)	<0.001	0.08	3.19 (10.66)	1.82
FTCT *	0.64 (0.14)	0.66 (0.14)	<0.001	0.17	0.02 (0.10)	3.03

Neuromuscular variables are presented as a mean (standard deviation). FMax = Maximal Force, TFMax = Time to Maximal Force, RFD = Rate of Force Development, IMP = Impulse, FTCT = Flight Time/Contraction Time, ES = Effect size, LOA = Limits of Agreement, %REF = percentage of the reference mean. * *t*-test, otherwise: Wilcoxon Test.

**Table 3:** Reliability parameters.

Variables	Lab	S&C	*p* Value	ES	ICC	SEM	CV	MD	MD%
FMax (N)	1710.42 (377.66)	1721.01 (404.81)	0.152	0.02	0.99	47.50	3.91	131.65	7.65
TFMax (ms)	543.48 (124.07)	542.56 (130.19)	0.887	−0.01	0.89	41.89	10.91	116.12	21.37
RFD (N·ms^−1^)	1.97 (0.81)	2.00 (0.85)	0.307	0.03	0.95	0.18	13.07	0.51	25.50
IMP (N·s)	173.44 (42.24)	173.31 (42.81)	0.782	0.00	1.00	3.01	2.45	8.33	4.80
FTCT	0.61 (0.17)	0.62 (0.16)	0.316	0.11	0.48	0.12	26.72	0.33	53.23

Neuromuscular variables are presented as a mean (standard deviation). FMax = Maximal force, TFMax = Time to maximal force, RFD = Rate of force development, IMP = Impulse, FTCT = Flight time/Contraction time, ES = Effect size, ICC = Intraclass correlation coefficient, SEM = Standard error of measurement, CV = Coefficient of variation (%). MD: Minimum difference to be considered real. MD%: Minimum difference to be considered real as a percentage of the highest mean (Lab or S&C).
